# Oral neurovascular hamartoma: An independent entity not associated to rhabdoid tumors

**DOI:** 10.4317/jced.62341

**Published:** 2025-01-01

**Authors:** Mario Guerrero-Martín, Guillermo García-Serrano, Laura Nájera-Botello, Adrián González-Jiménez, Kora Sagüillo-Pallares, Mario Pezzi-Rodríguez, Julio Acero-Sanz

**Affiliations:** 1 Oral and Maxillofacial Surgery Department, Ramon y Cajal and Puerta de Hierro University Hospitals, Madrid, Spain; 2 Pathology Department, Puerta de Hierro University Hospital, Majadahonda, Spain; 3 Prof and Head of Oral and Maxillofacial Surgery Department, Ramon y Cajal and Puerta de Hierro University Hospitals, Madrid, Spain

## Abstract

**Background:**

Oral cavity neurovascular hamartomas are extremely rare, with unknown prevalence, possibly due to their rarity, unrecognition or under-report. We describe a 6-year-old female patient with this uncommon lesion and review existing literature.

**Results:**

The patient presented with asymptomatic papular lesions, which increased in size and number since birth. First, one of the papular lesions on the lower lip was excised for histopathologic study, revealing the unexpected diagnosis.

**Discussion:**

Oral neurovascular hamartomas are unusual and challenging lesions to diagnose. Even though their benign nature, the need of differential diagnosis with other oral lesions makes necessary their histopathological study and confirmation.

**Conclusions:**

Even oral neurovascular hamartoma is an uncommon entity, surgeons and oral pathologist should be aware of this entity in order to distinguish it from other lesions. Besides, it is not clearly related to rhabdoid tumors as other authors have stablished previously, being an independent entity with great prognosis for the patient.

** Key words:**Oral neurovascular hamartoma, benign tumors, pediatric age masses, rhabdoid tumors.

## Introduction

Oral neurovascular hamartoma is an uncommon entity, with only 25 cases reported worldwide to our knowledge. Most common sites of appearance are the tongue (54%), buccal mucosa (17%) and lower lip (17%) (1). In contrast with other type of hamartomas that are usually associated to syndromic conditions as PTEN mutations, Tuberous Sclerosis or Cronkhite-Canada syndrome, this lesion has always been reported as a sporadic entity (2). Usually reported as solitary exophytic or ulcerated lesions, they often mimic other hamartomatous, infectious or neoplastic proliferations (3). Our objective is to report a new case of a 6-year-old patient diagnosed of oral neurovascular hamartoma affecting the lower lip, to compare it with the existing literature and to contribute to stablish it as an independent lesion non-associated with rhabdoid tumors. Histopathologic and immunohistochemical features are discussed and

a review of the current literature is conducted.

## Case Report

**Figure 1:**
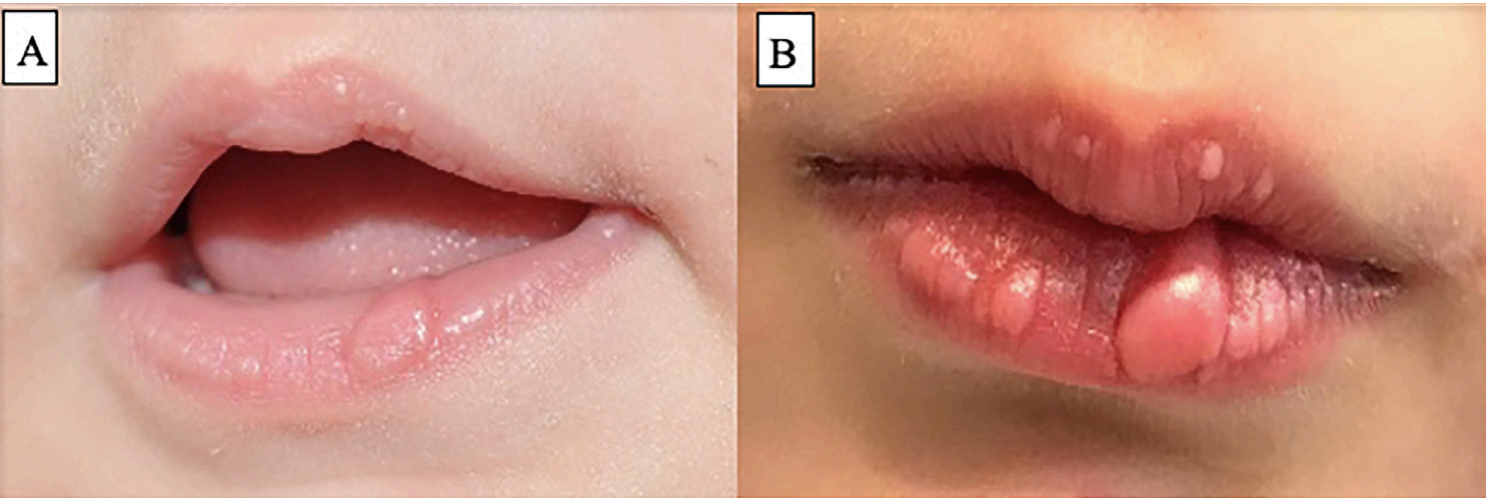
Clinical aspect of lesions when patient is 1-year-old (A) and 6-year-old (B).

A 6-year-old female child presented at our outpatient clinic with asymptomatic papular lesion on lower lip, present since birth, but with an increase in size and appearance of another two lesions in the same area. No previous diseases were described. Physical examination revealed two mucosal soft masses in lower lip, well delimited, not indurated (Fig. 1). Mobility of lower lip was not affected.

Ultrasound examination showed three poorly delimited lesions on the vermilion border of lower lip, not infiltrative, measuring 2.1 x 1.5 cm the biggest one, indicative of benign lesion to determine under pathological examination.

**Figure 2:**
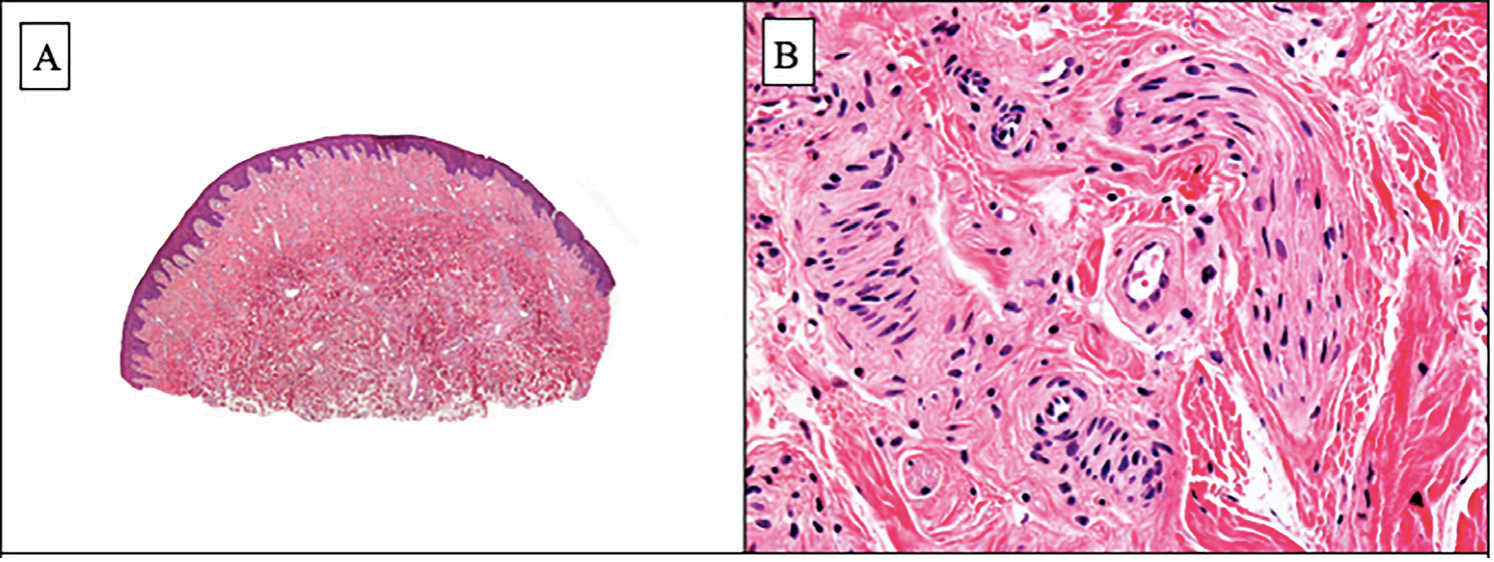
A. Scanning power showing lightly hyperplastic epidermis and numerous vascular structures in the dermis. B. Higher magnification showing abundant enlarged nerve fibers around blood vessels.

**Figure 3:**
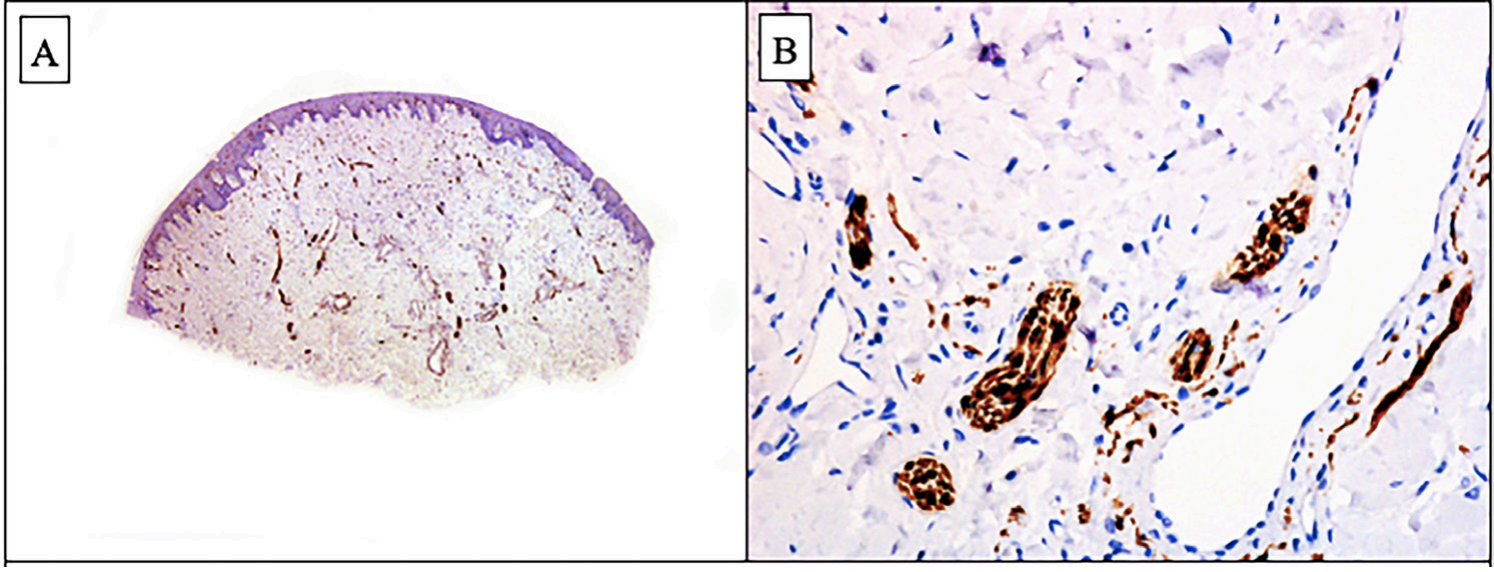
Immunohistochemical stain for S100 protein: A. Scanning power showing numerous S100 protein positive structures in the dermis. B. Higher power showing numerous mature S100 protein positive mature nerve fibers.

One of the papular lesions on the lower lip was excised for histopathologic study. It demonstrated that beneath a normal epithelium, the corium of the lower lip was involved by numerous vascular structures of variable thickness walls and numerous mature nerve fibers surrounded by perineurium. These two components were embedded in a fibrillary collagen and myxoid stroma (Fig. 2). Immunohistochemical studies showed that the vascular structures we lined by a thick smooth muscle actin (SMA) positive wall and a monomorphous layer of CD31-positive endothelial cells. Nerve fibers showed S100 protein positivity and a peripheral layer of EMA-positive perineurial cells (Fig. 3). Once analyzed, the other lesions were also excised.

## Discussion

Oral neurovascular hamartoma is an extremely rare entity to diagnose in our daily clinical practice. Allon *et al*. reported only 25 cases around the World, 11 in men and 14 in women, with ages between 6-76 years, mean 44. It usually presents as a painless exophytic mass with pinkish or yellowish coloration, pediculated or wide based, measuring 0.25-2.5 cm. Most common site of appearance is the tongue, followed by buccal mucosa and lower lip. Histopathologically, the lesion consists of a haphazard mixture of well-formed nerve fibers and blood vessels in a loose matrix, with minimal or absent associated inflammation (1,3).

The appearance of epithelial or mesenchymal hamartomas in the oral cavity is very uncommon, thus, the presence of neural o neurovascular components is even more unusual. Vascular hamartomas are the most frequent hamartomas in head and neck. Histopathologically, they are non-encapsulated and poor circumscribed from normal tissue (3).

As the age of presentation is quite wide, this cannot be criteria to diagnose oral neurovascular hamartoma. The differential diagnosis of this lesion includes reactive lesions. Compared to fibrous hyperplasia, our described entity presents more nerve bundles and less blood vessels. The painless or sensitive symptoms can help to distinguish it from traumatic neuroma. Mucosal neuromas consist of a better demarcated proliferation of mature nerve fibers surrounded by perineurio and they lack of the vascular component characteristic of neurovascular hamartoma. The presence in oral neurovascular hamartoma of these neural components makes also the difference from benign mesenchymoma (1).

Treatment of oral neurovascular hamartoma is based in complete surgical resection, although if poor cosmetic or functional outcome is foreseen, partial resection may be indicated. Histopathological confirmation is necessary due to the ability of mimicking aggressive lesions, which may make patients undergo inadequate treatments (4). Resection including the whole lesion is curative; however, incomplete excision may cause recurrence. Close post-surgical follow up is strongly recommended (3).

Finally, in our opinion, it should be noted that term neurovascular hamartoma has been also used to name of completely different entity. Perez Ataide *et al*. in 1994 reported two cases of a congenital, benign, probably hamartomatous, lesion of the upper dermis in two children who subsequently developed malignant rhabdoid tumors (5). The dermal lesions, which they named “neurovascular hamartomas”, were characterized by a proliferation of capillaries in a background of bland spindle cells with possible neural features. In one patient developped the malignant rhabdoid tumor in the kidney, and a synchronous primitive neuroectodermal tumor of the central nervous system was the cause of his death. The second patient had two neurovascular hamartomas, and a malignant rhabdoid tumor arose in contiguity with the deepest portion of the larger of the two hamartomas. The authors concluded that this congenital benign dermal lesion should be considered as a cutaneous marker of malignant rhabdoid tumors.5 In our opinion, this cutaneous marker of malignant rhabdoid tumor should be named with a different term, because the authentic neurovascular hamartoma of the oral cavity was described before and it is an entirely benign lesion with no any associated disorder.

## Conclusions

Despite the rarity of oral neurovascular hamartoma, it is necessary to include this entity in the differential diagnosis of oral masses. Most importantly, they are independent benign lesions no related to rhabdoid tumors. The awareness of its clinical presentation, diagnosis and correct treatment is essential.

## Data Availability

The datasets used and/or analyzed during the current study are available from the corresponding author.
